# Perceptions of northeast Thai breastfeeding mothers regarding facilitators and barriers to six-month exclusive breastfeeding: focus group discussions

**DOI:** 10.1186/s13006-018-0148-y

**Published:** 2018-04-05

**Authors:** Thiwawan Thepha, Debbie Marais, Jacqueline Bell, Somjit Muangpin

**Affiliations:** 1 Department of Advanced Midwifery, Faculty of Nursing, Khonkaen University, 123 Mittapap Road, Khonkaen, Thailand; 2 Warwick Medical School, University of Warwick, Coventry, UK; 3 College of Life Sciences and Medicine, University of Aberdeen, Aberdeen, UK; 4 Department of Advanced Midwifery, Faculty of Nursing, Khonkaen University, Khonkaen, Thailand

**Keywords:** Facilitator, Barrier, Six-month exclusive breastfeeding, Social media, Northeast Thailand, Focus group discussion

## Abstract

**Background:**

The 6-month exclusive breastfeeding rate in the Northeast region of Thailand has recently significantly decreased in contrast to all other regions in Thailand. The factors that have influenced this decrease remain unknown. Hence, it is suggested that an investigation into factors that could improve or hinder EBF for 6 months in Northeast Thailand may be required to inform the development of relevant interventions to improve this situation. This study aimed to identify perceived facilitators and barriers to providing exclusive breastfeeding for 6 months in Northeast Thailand among breastfeeding mothers.

**Methods:**

Six focus group discussions were conducted with a total of 30 mothers aged 20 to 40 years who had children aged between 4 and 6 months and were currently breastfeeding or had breastfeeding experience. Participants were recruited through self-selection sampling from Khonkaen hospital (urban), Numphong hospital (peri-urban) and private hospitals (urban) in Khonkaen, Thailand. Thematic analysis was employed to analyse the data.

**Results:**

Five main themes, with 10 sub-themes, were identified as either facilitators (+) or barriers (−), or in some cases, as both (+/−). Breastfeeding knowledge, perceptions, maternal circumstances, support, and traditional food were the main identified themes. Mother’s breastfeeding knowledge, intention to breastfeed, and social media were perceived as facilitators. Perceptions, employment, and formula milk promotion were perceived as barriers. Family, healthcare, and traditional food were perceived as both facilitators and barriers. The perception that social media was a way to access breastfeeding knowledge and support mothers in Northeast Thailand emerged as a new facilitating factor that had not previously been identified in Thai literature relating to facilitators and barriers to exclusive breastfeeding. Intention to breastfeed, family support, healthcare support and traditional food were mentioned by all groups, whereas mothers from urban areas specifically mentioned mother’s breastfeeding knowledge, social media and employment sub-themes. Only mothers from the peri-urban area mentioned formula milk promotion and only mothers who had delivered in public hospitals mentioned the perceptions sub-theme.

**Conclusions:**

Knowledge about these facilitators and barriers may inform the design and development of specific and relevant interventions to improve the 6-month exclusive breastfeeding rate in the Northeast region of Thailand and be useful in other contexts. Social media emerged as a newly perceived facilitator in the Thai context and may be a useful inclusion in a 6-month exclusive breastfeeding intervention model.

**Electronic supplementary material:**

The online version of this article (10.1186/s13006-018-0148-y) contains supplementary material, which is available to authorized users.

## Background

Since 2001, 6 months of exclusive breastfeeding (6-month EBF) has been recommended [1] and a global target of 50% by 2025 has been set [2, 3]. The National Economic and Social Development Board of Thailand (2012) set a goal of 6-month EBF (30% of infants) at the 11th National Economic and Social Development Plan of Thailand 2012–2016 [4]. It can clearly be seen that, both internationally and in Thailand, 6-month EBF has been identified as a priority and is of public health concern.

In Thailand, since 1989, many EBF initiatives, such as setting the National Breastfeeding Strategy, the Baby-Friendly Hospital Initiative, the Thai Breastfeeding Center Foundation, the Family Relationship Project, and subscribing to the International Code of Marketing for Breastmilk Substitutes, have aimed to protect, promote and support 6-month of EBF [5–7]. However, despite these initiatives, the 6-month EBF rate in Thailand dropped from 15.0% in 2009 to 12.3% in 2013 and is far from reaching the national target [8, 9]. Among the five regions of Thailand, the 6-month EBF rate in the Northeast region was the only one to show a significant decrease from 26.9% in 2009 to 13.8% in 2013, while in all the other regions the rate increased slightly [8, 9] (Table 1). Therefore, the Northeast region of Thailand should be of specific concern and a focus area for interventions that aim to improve six-month EBF rates. Reasons for the drop in the rate of 6-month EBF in Thailand have not been identified. However, the facilitators and barriers to EBF in Thailand have been reviewed [10] and show that factors related to the mother, family support, situation and social context, infant factors, and healthcare profession and healthcare service influence EBF in Thailand. To inform the design and development of effective and relevant interventions for this region, it is important to explore the perceptions of mothers regarding specific facilitators and barriers that may be impacting on mothers’ ability to continually exclusively breastfeed for 6 months. Consequently, the aim of this study was to explore the perceptions of Northeast Thai breastfeeding mothers regarding facilitators and barriers to 6-month EBF.

**Table 1:** Six-month EBF rate in Thailand from 2009 to 2013

Year	Six-month EBF rate (%)						Author
	Whole Kingdom	Bangkok	Central	North	Northeast	South	
2009	15.1	1.7	6.1	9.4	26.9	10.4	NSO Thailand, 2010
2013	12.3 ↓	8.2	7.9	19.6	13.8 ↓	12.2	NSO Thailand, 2013

## Methods

Khonkaen province was purposively selected for the study setting as it includes one of the major cities of Northeast Thailand. Khonkaen is approximately 400 km north of Bangkok with a population of nearly 2 million people and a birth rate of 11.84 per 1000 people in 2012 [11]. Mothers were recruited from three different settings within Khonkaen province to include mothers from urban and peri-urban areas as well as mothers who had delivered at public and private hospitals. The catchment areas were an urban public tertiary hospital (Well Baby Out Patient Department, Khonkaen Hospital), a peri-urban public secondary hospital about 35 km north of Khonkaen (Numphong hospital), and three private hospitals in Khonkaen. These five hospitals provide standard healthcare for the mother and infant, such as antenatal education including breastfeeding, growth monitoring of the infant and immunisations at 2, 4 and 6 months.

As perceptions and experiences were being investigated, a qualitative approach using focus group discussions (FGDs) was followed. FGDs have been shown to be an efficient methodology in terms of time and resources, to explore and clarify the perceptions of participants [12]. This data collection method enables the researcher to access a range of ideas due to the interaction between participants, without participants feeling pressured [12]. The eligibility criteria for participants were: Northeast Thai mothers aged 20 to 40 years, both primiparous and multiparous, with children aged between 4 and 6 months, who were currently breastfeeding this child or had prior breastfeeding experience. Mothers who had never initiated breastfeeding were excluded as the barriers to initiation were not being explored in this study.

Participants were selected using self-selection sampling. Recruitment posters (in Thai) were placed at each of the selected hospitals on notice boards. Willing prospective participants, who contacted the researcher, were asked to select a suitable date from a list of possible FGDs at one of three central locations - private rooms at Khonkaen and Numphong hospitals or a meeting room at the Faculty of Nursing, Khonkaen University. Approximately 1 month before the FGDs were scheduled, information sheets (in Thai) were sent to participants via post or email in accordance with their expressed preference.

Participants willingly signed consent forms prior to the start of each FGD, which comprised three to six participants. They were conducted in Thai, took approximately 1 h, and were conducted until the point of data saturation. Data saturation is achieved when data collection and analysis results in no further categories or themes being identified [13]. The primary investigator (PI), acting as the facilitator, followed a topic guide which was based on evidence from a review identifying facilitators and barriers to EBF in Thailand specifically [10]. Firstly, the ground rules were specified, followed by participants introducing themselves and saying a little about their children. Participants were then asked how long they intended to exclusively breastfeed or had exclusively breastfed, leading to the main questions relating to what helped them to continue exclusive breastfeeding and what hindered this. Prompts concerning aspects related to the mothers, babies, health professionals and social conditions were used if not raised. The topic guide was piloted with nursing students and staff at the Khonkaen University. At the start of each FGD, the facilitator confirmed the meaning of the term 6-month EBF with all the participants, ensuring that everyone understood the WHO definition. An observer made notes and ensured that the session was audio-recorded. Participants were not offered any incentives, but refreshments were offered at the end of the discussion. The recorded FGD were transcribed verbatim in Thai and then translated into English. The transcriptions were back-translated to check the accuracy of translation. Thematic analysis was used to analyse data and develop categories and themes of facilitators and barriers to 6-month EBF in Northeast Thailand by the PI (TT) and independently by a second reviewer (DM). The independent analysis followed the recognized steps of familiarization with the data, generation of initial codes, identification of themes among codes, review of themes and defining and naming themes [13]. Verification of the data and themes was conducted using member checking and peer debriefing with the other members of the research team (JB, SM). The frequency of comments related to each theme were calculated to determine whether there were any unique sub-themes for urban vs peri-urban dwellers and those that had delivered at private vs public hospitals. Ethical approval was granted by the College Ethics Review Board (CERB), University of Aberdeen (No. CERB/2015/3/1147) as well as the Khonkaen Hospital Ethics Committee, Thailand (No. KE58059).

## Results

**Fig. 1:**
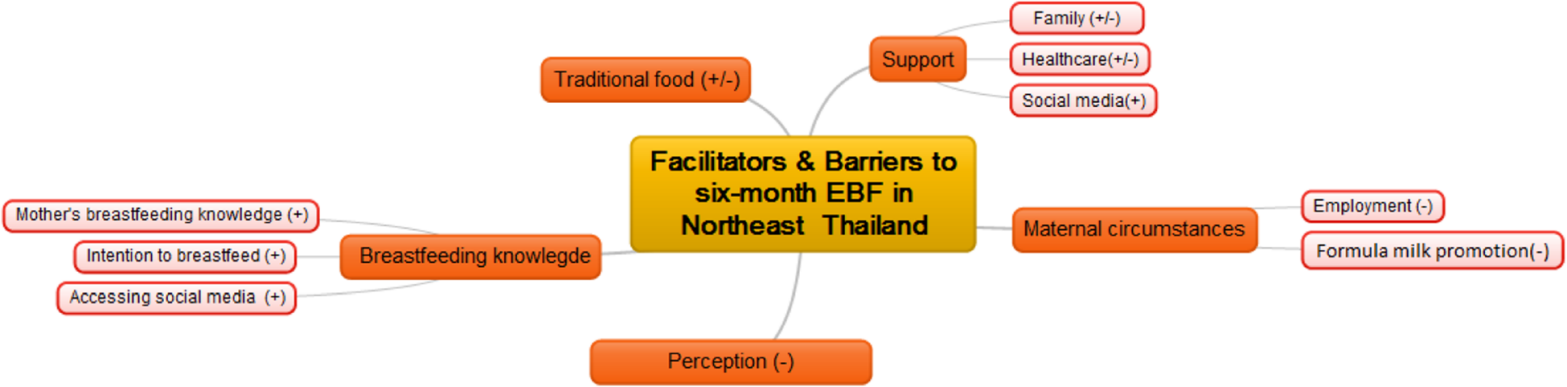
Facilitators and barriers to 6-month EBF in Northeast Thailand

Six FGDs comprising a total of 30 participants were conducted in August 2015 (Additional file 1: Table S1). Participants had a mean age of 30 years (SD = 5.49), were engaged in various occupations and encompassed a wide educational range, from high school to master’s degree level. A range of settings were represented, with mothers recruited mainly from urban (80%) but also peri-urban areas (20%), and had mainly delivered at public (73%) but also at private (27%) hospitals. Data saturation was achieved by the completion of these six FGDs. Five themes, with 10 sub-themes were identified as either facilitators (+) or barriers (−), or in some cases as both (+/−). The themes were named as breastfeeding knowledge, perceptions, maternal circumstances, support and traditional food (Fig. 1). Illustrative quotes are provided anonymously from participants for each theme, but are individually identified according to which FGD they attended e.g. the second participant in the fifth FGD would be 5B.

### Breastfeeding knowledge

Breastfeeding knowledge included three sub-themes namely mother’s breastfeeding knowledge, intention to breastfeed, and accessing social media for breastfeeding information. Participants suggested that knowledge about the advantages of breast milk, including reducing infants’ susceptibility to infection, its rich nutrient content, and availability without cost, could facilitate 6-month EBF, by initially stimulating mothers, and encouraging them to continue with EBF for 6 months. Here is a selection of responses to the prompt “Why did you exclusively breastfeed your child?”. This sub-theme was specifically mentioned by mothers from urban areas (Table 2).

**Table 2:** The frequency of comments related to each theme, identifying unique sub-themes for urban vs peri-urban dwellers and delivery at private vs public hospitals

Sub-themes	Khonkaen hospital	Private hospital	Numphong hospital	Total
Mother’s BF Knowledge (+)	12	8	0	20
Intension to BF (+)	4	4	1	9
Accessing social media (+)	5	5	0	10
Perception (−)	1	0	1	2
Employment (−)	12	5	0	17
Formula milk promotion (−)	0	2	0	2
Family (+/−)	9	6	2	17
Healthcare (+/−)	4	7	3	14
Social media (+)	3	0	0	3
Traditional food (+/−)	9	9	4	22
Total	59	46	11	116

Urban dwellers = Khonkaen and private hospital groups

Peri-urban dwellers = Numphong hospital group

Delivered at a private hospital = Private hospital group

Delivered at public hospital = Khonkaen and Numphong hospital

*“Breastmilk has immunology. It is good for my child so I continue to breastfeed”5B*

“Breastmilk is filled with all (the necessary) nutrients. No need for other food and new mothers should know that it (breastfeeding) saves on cost.” 1C

*“Drinking breastmilk does not cause obesity. Formula milk makes children fat.”* 4E

Participants were concerned about the practicalities of milk supply for continuing EBF. Participants indicated that mothers who understood about breast milk production and expressing tended to continue exclusively breastfeeding for longer.

“*I know that more suckling (results in) more breastmilk that is what made me continue breastfeeding.” 1D*

“I can express breastmilk 8 times - 1 day is 24 hour – so I pump every 3 hours. Then my breastmilk became a lot. When breastmilk became a lot, I had milk to continually breastfeed. This led me to have a long period of breastfeeding” 5B

Participants felt that an intention to EBF could motivate them to overcome any obstacle to breastfeeding, if they had the right intention at the start. Mothers from all areas mentioned this sub-theme (Table 2).

*“There are many advantages of breastmilk, I wish my son to get these, so I try to carry on with exclusive breastfeeding.” 6C*

“If we intend to exclusively breastfeed, it will be a success.” 1C

*“(I) intended to (exclusive breastfeed) therefore I can do it” 2F*

*“The intention of the mother is important for exclusive breastfeeding” 5C*

Social media, including Facebook and web pages, was defined by participants as a way to access breastfeeding knowledge. Participants reported utilising internet-based social media to gain information for solving breastfeeding problems. The problems usually related to how to express and maintain breast milk supply. Most mothers perceived social media as a peer support platform for breastfeeding mothers. This sub-theme was specifically mentioned by mothers from urban areas (Table 2).

“I searched for and shared information on the (Facebook page) where mothers have a little breastmilk club (Kun-mai-nom-noi) and one for mothers who express breastmilk (Kun-Mai-nuk-pump). Mothers from all countries share information and I got inspiration from the Facebook group to continuously breastfeed even though I had a breastfeeding problem.” 5B

“When I took maternity leave, I joined the mothers’ (Facebook) group (Pumping mothers who do everything for their child group). I joined to search for information. Many people submitted questions and they had responses from the group. The way to store breastmilk was shown; small points were shown … it stimulated others …if others could do it, so I tried my best.” 6B

*“I browsed the internet for breastfeeding information” 4D*

“I just watched YouTube, browsing the internet.” 3A

*“Sometimes, (I) searched on web sites.” 5A*

*“I just asked people around me and browsed the internet.” 4D*

“(I browsed about review of breastfeeding) On the internet. On the Pantip website (Pantip is a popular Thai-language website and discussion forum relating to any issues).” 1A

*“(I) searched on Google and listened to other people.” 4F*

### Perceptions

Mothers reported a perception that baby boys need more breastmilk than baby girl. They felt this may be a barrier to 6-month EBF as mothers with boys may be more likely to stop breastfeeding them before 6 months if they felt their milk supply was insufficient. This sub-theme was specifically mentioned by mothers who had delivered in a public hospital (Table 2).

“My second child is a boy; he needs more breastmilk than his older sister (needed). My breastmilk is not enough for him so I started giving formula milk along with my breastmilk” 4B

“My child is a boy. He needs a lot of breastmilk.” 2F

### Maternal circumstances

The theme of maternal circumstances includes sub-themes related to employment and formula milk promotion. Mothers felt that full-time working did not allow sufficient time for breast milk expression, which led to the reduction of breast milk production. Also, the lack of private places to express breast milk in the workplace was mentioned as a barrier. Less supportive supervisors at work were also recognised as a barrier to 6-month EBF. Mothers who were working reported being stressed during work if their supervisor did not give them the opportunity to breastfeed or express milk. In addition, many working mothers suggested that maternity leave should be extended to 6 months from the current level of 3 months. Mothers from urban areas specifically mentioned this sub-theme (Table 2).

“I pumped every 3 hours at home, but (I) pumped every 4 to 5 hours at work because I need to concentrate with my job. This lead (to) the reducing of the frequency of pumping. It reduced the amount of breastmilk” 4C

“I finish working quite late. The work is very busy. It is not convenient to pump breastmilk during working time (in my situation).” 4D

“In other occupations, for example woman workers in a factory, nurses etc., there should be breastfeeding places provided. Even though I have a room available to me I feel a bit of anxiety; I feel uncomfortable and I also get stressed when I do breast pumping. It’s an obstacle to breastfeeding” 6C

“Their boss does not understand why the mother takes 25–30 minutes for expressing breastmilk. If mothers cannot express, breastmilk will decrease. Finally, the mother will need to stop breastfeeding.” 5B

“If mothers can take maternity leave for 6 months, it will be nice. There may not be any problems and (the) mother can only concentrate on breastfeeding until her baby is 6 months” 3A*.*

“At 4th month, I came to work in Khonkaen. I did not know how to stimulate (milk production). I pumped breastmilk and kept it in the refrigerator but I did not know the best way to pump breastmilk. My child stayed in my hometown in another province (Ka-la-sin province). My breastmilk became less and less and then it totally disappeared. I thought that if I could have taken maternity leave for 6 months, my breastmilk may have been enough for my child.” 6C

The promotion of formula milk was mentioned as a barrier to breastfeeding initiatives and successful EBF. The strategies to promote formula milk by companies varied. For example, some companies offered free formula milk to hospitals or mothers, especially in private hospitals. Some mothers who delivered at private hospitals reported that they had been given free formula milk when they were discharged. The attractive advertisements on public media, including television channels, also distracted mothers from their intention to continue EBF. This sub-theme was specifically recognized by mothers who had delivered at a private hospital (Table 2).

“In private hospitals, formula milk companies sponsor the hospital so they provide formula milk for the infant. If I do not have breastmilk, I may feed formula milk to my child” 5A

### Support

Support had three sub-themes, namely support from family, the healthcare system and social media. These were often seen as both facilitating or as a barrier. Some mothers stated that their family was very helpful; they took care of the baby, provided financial support, and gave suggestions to support them while breastfeeding. Mothers from all groups mentioned this sub-theme (Table 2).

“My grandmother helped me to take care of the baby, I just had to be concerned about exclusively breastfeeding” 1B

“If family support me such as do housework or provide financial support, it will support exclusive breastfeeding; (so that) I can (have time to) hug, hold (my child) and nurture the relationship.” 3A

“After I went back to work, my grandmother helped me by feeding my child with my defrosted breastmilk.” 3D

“My husband is nice; he said that I don’t need to work; just take care of the child. So I am not worried about anything, not stressed and I had more time to breastfeed my child” 2F

In contrast, some mothers reported that members of the family such as the husband, their previous child, and grandparents, can have a negative influence on their breast milk production and hence on 6-month EBF. Husbands can cause mothers to feel stressed and some mothers experienced older children often exhibiting disruptive behaviour while mothers were breastfeeding their younger child. A few mothers also reported that elders provided conflicting advice.

“Sometimes I am moody because of my husband. I got stress from my husband then my breastmilk is poor in releasing and I do not feel like breastfeeding anymore” 1A

“My eldest son who is 1 year and 9 months (old) is secretly jealous of the youngest child; when the youngest child has breastmilk, there would be a strange situation in that he would like to sleep on my lap; it's a pity. It is an obstacle while I breastfed” 5C

*“Grandmothers and people who have experience in taking care of children recommended that I should give Se-ly-lax [supplementary food] to my child before he was 6 months (old).”4E*

In terms of facilitators, available and accessible health care services were identified as facilitators to successful 6-month EBF. Support from health care personnel, especially from nurses, was also mentioned as helping women to overcome their problems or concerns. Many mothers said that breast milk clinics and hotlines were available, which enabled them to consult a healthcare professional easily and immediately when they had a problem with breastfeeding. Health volunteers, trained by the primary healthcare system, also worked and supported breastfeeding mothers in the community. The policies of government hospitals in Northeast Thailand support EBF by allowing the mother and infant to be together after birth enabling the initiation of breastfeeding. They also prohibit the promotion of formula milk. This sub-theme was noted by mothers from all groups (Table 2).

“I am confident in the [EBF] knowledge that the nurse at the government Hospital taught me; they also have a telephone service for mothers. If anyone has insufficient milk, they can call them; if you want to take medicine, you can call them 24 hours; so I am not worried. It made me confident to breastfeed” 4A

“The healthcare volunteer was the person to teach me about breastfeeding and visited me while I was on maternity leave. It made me confident to exclusively breastfed” 2B

*“In government hospitals, if the mother and child are in good health, they will be advised to start exclusive breastfeeding immediately after birth. I had a chance to breastfeed my child immediately*” *5A*

“I compare with my sister who delivered in government hospital. They did not provide formula milk.” 5C

In contrast, mothers felt that the policies of private hospitals can create negative factors. A clear distinction emerged in terms of experiences at private versus governmental hospitals. Mothers who delivered at private hospitals indicated that they would be separated from their baby for at least 6 h following delivery. A lack of knowledge about EBF among some health care professionals was also reported as a barrier to 6 months EBF.

“[when I delivered at a private hospital 5 years ago], my baby and I were separated -for 24 hours] Nowadays, a baby is separated and closely observed for at least 6 hours at another unit..Even though the amounts of separating hours has decreased, it’s a barrier to the first breastfeed in the first hour after the child’s birth.” 5A

“I did not feed formula milk to my first child because the nurse taught me that the size of a child’s stomach is quite small so it is not necessary to add more formula milk. If I was not told, I would have thought that my child is not full; so I may have given the child formula milk. For my second child, he (the doctor) did not encourage breastfeeding; he gave a few suggestions but I heard that my friend who had delivered at this hospital had not been successful (in exclusive breastfeeding).” 4A

Social media was reported as a facilitator to 6-month EBF. Mothers reported that they not only got breastfeeding knowledge, but also got encouragement from other mothers in social media groups such as Facebook. This sub-theme was specifically mentioned by mothers from urban areas (Table 2).

“When I took maternity leave, I joined the mothers’ (Facebook) group (Pumping mothers who do everything for their child group). I joined to search for information. Many people submitted questions and they had responses from the group. The way to store breastmilk was shown; small points were shown … it stimulated others …if others could do it, so I tried my best.” 6B

“There is a (Facebook) group that has the same objective; encouraging everyone; helping to ensure breastfeeding over a long period.” 6A

### Traditional food

According to Thai tradition, mothers believed that drinking hot water, boiled herb (ginger), and having banana blossom and ginger could stimulate breast milk production and therefore facilitate 6 months EBF. However, mothers also believed that eating sweet and oily food may suppress breastmilk production and therefore act as a barrier. Mothers from all areas mentioned this sub-theme (Table 2).

*“You need to have Thai herbs, ginger and stir fried banana blossom curry. These can lead to a lot of breastmilk which encouraged me to breastfeed” 2E*

“Drink hot water and ginger – (it) can produce a lot of breastmilk” 3E

“If (I) eat sweet and oily food, breast milk does not come out. It made me stop breastfeeding” 3C

## Discussion

The perceptions of Northeast Thai breastfeeding mothers regarding facilitators and barriers to 6-month EBF were explored via FGDs. Thai mothers reported that mother’s breastfeeding knowledge, intention to breastfeed, and social media were perceived to be facilitating factors to continuing to exclusively breastfeed for 6 months. Intending to EBF was noted by all groups in the study indicating that intention can be inspirational to initiate and continue EBF for 6 months. The literature confirms that others have found that the intention of the mother to breastfeed facilitates a longer breastfeeding duration [10, 14]. Although studies in other contexts have also shown that mothers’ breastfeeding knowledge facilitates 6-month EBF [10, 15–18], it is interesting that this was mentioned, often, by urban dwellers only in this study. This should be interpreted carefully though as the data is qualitative [12, 13]. The fact that some factors were unique to specific groups though is important to take into consideration for developing specific and relevant interventions. It seems that a multi-pronged approach may be most appropriate with some interventions being focused on specific groups such as in urban areas or at private hospitals. This phenomenon has been explored and may explain why some interventions are less effective in different settings [19–21]. Related to the findings about mother’s BF knowledge, mothers who lived in urban areas and were further educated, also uniquely reported using social media to access breastfeeding knowledge, and provide or receive group or peer support to encourage continuation of EBF. On the one hand, this finding was surprising as it had not previously been reported in Thai literature [10], but on the other hand it is not surprising with the global trend of increasing digital literacy. There has been a steady increase in internet users with 40% of people in Thailand reportedly able to access the internet in 2016 [22] and the number of social media users has grown rapidly with 2.3 billion people in the world [23] and 56% of Thai people using social media [24]. The use of social media for business, political and healthcare purposes has been reported [25, 26] and there is growing evidence that using social media (such as Facebook, online support groups and web pages) is becoming a popular intervention for support, information dissemination, exchanging information or experience among group members and is having a positive impact on health status [27]. This may therefore be another intervention that would be best targeted to specific groups within the region.

Identified barriers, namely perceptions, employment, and formula milk promotion, were consistent with barriers described in the global literature [28–31]. Our findings show that some Northeast Thai mothers who had delivered at a public hospital perceived that boys need more breast milk than girls. This phenomenon has also been reported in Zimbabwe [28], although where the delivery took place was not noted specifically. In Laos, for example though, the gender of an infant has not been related to continue breastfeeding [29] indicating that this is a topic for further investigation before including it in an intervention model. As reported for some of the facilitators, the perceived barriers of employment and formula milk promotion were mentioned by mothers from urban areas only. Various studies have also found a lack of workplace support [32] and stress at work as a barrier to 6 months EBF in Thailand [10]. Brien, Buikstra and Hegney [30] showed that stress affected the duration of breastfeeding. The short duration of maternity leave was also found to be barrier to EBF in Malayasia, Pakistan, India and central Thailand [10, 31–34]. The working environment should therefore be a focus for interventions for mothers in urban areas specifically, whereas lobbying government for longer maternity leave should be considered in Northeast Thailand. Finally, the fact that mothers reported that the promotion of formula milk, including free samples from private hospitals, may be a barrier to EBF, has also been reported in India, where the aggressive promotion of formula milk was identified as a barrier [31, 35]. It seems that raising awareness about the promotion of formula milk could be another targeted intervention to increase 6-month EBF rates, especially at private hospitals in Northeast Thailand.

All Northeast Thai mothers perceived that family and healthcare support and traditional food can be both a facilitator and a barrier. The family (husbands and family members) have also been identified as facilitators by other studies. Mothers have reported that husbands [34] and other family members can help them take care of the infant and offer psychological support [28, 36, 37]. However, grandmothers can present a barrier which is similar to findings in a study conducted in Pakistan [16]. In Thai culture, grandmothers and husband often live with the family providing psychological and financial support [38]. Interventions should therefore focus on educating everyone in the family who provide support. Many studies have confirmed that a wide variety of services, including 24-h counselling and hotlines, and accessible healthcare support services, facilitate 6-month EBF [10, 39, 40]. In terms of healthcare professionals’ knowledge, one study has found that the breastfeeding knowledge of nurses is positively related to the rate of breastfeeding initiation [41]. In contrast, health professionals sometimes offer contradictory advice [41] making it important to ensure that messages are consistent when training is provided to healthcare professionals. In Colorado, Weddig, Baker, and Auld [41] found that the hospital lactation policies (formal and informal), which had not been updated recently, were reported as a barrier to breastfeeding but that “the rooming in” policy (parents keeping their newborn with them 24 h a day) was found as a facilitator in some hospitals in America. Educating healthcare professionals may therefore not be enough, but ensuring that policies that protect, promote and support BF are in place and implemented is also important to an intervention strategy for Northeast Thailand. Finally, traditional foods were reported as both facilitators and barriers by Northeast Thai mothers. Many traditional foods have been reported in terms of certain foods and drinks stimulating or reducing breastmilk production. The specific foods and drinks differ by context, with the Thai belief being that consumption of hot water, ginger, boiled herbs, and banana blossom can stimulate breastmilk production. Some of these beliefs have some evidence such as ginger being recognized as a promising natural galactagogue which can improve the amount of breastmilk [42]. In Zimbabwe, traditional beliefs are that foods such as amahewu, salted nuts, tea, and juices stimulate breastmilk production [28].

### Study strengths and limitations

The main strength of the current study was that it included mothers from urban and peri-urban areas in Northeast Thailand, mothers of various ages, employment and educational backgrounds. This provided a wide range of perspectives of mothers in Northeast Thailand, but the small and purposive sample does limit the generalisability of the findings [13]. Using a qualitative approach allowed for a deeper understanding of the perspectives of the major stakeholders, namely breastfeeding mothers, regarding this topic. Such data will be useful to inform the development of relevant interventions to improve 6-month EBF rates in this region. A limitation of this study is that mothers who did not breastfeed were not included, therefore the factors that influence women in their initial decision about breastfeeding were not explored, however this was not the focus of this study.

## Conclusions

In this study, the specific facilitators and barriers to 6 months EBF in Northeast Thailand were explored. Five themes were identified as facilitators and barriers to continued 6-month EBF. Mother’s breastfeeding knowledge, intention to breastfeed, social media were reported as facilitators. Barriers included perceptions, employment, and formula milk promotion. Family, healthcare and traditional food were identified as both facilitators and barriers. Support provided by social media, not previously reported in Northeast Thailand, was identified as a possible facilitator. These findings may inform the development of relevant 6-month EBF interventions in Northeast Thailand.

## Additional file

Additional file 1: **Table S1.** Characteristics of participants involved in the six FGDs. (DOCX 29 kb)

## Abbreviations

BD: Bachelor’s degree
DM: Debbie Marais
EBF: Exclusive breastfeeding
FGD: Focus group discussions
HS: High School
JB: Jacqueline Bell
MD: Master’s degree
PI: Principle investigator
SD: Standard deviation
SM: Somjit Muangpin
TT: Thiwawan Thepha
WHO: World Health Organization
