# Supplementary material for: Perceptions of northeast Thai breastfeeding mothers regarding facilitators and barriers to six-month exclusive breastfeeding: focus group discussions
Source: Int Breastfeed J. 2018 Apr 5;13:14. doi: 10.1186/s13006-018-0148-y (PMC5885310; doi:10.1186/s13006-018-0148-y)
Supplement: Supplementary file 1 — Table S1. Characteristics of participants involved in the six FGDs. (DOCX 29 kb) [file 13006_2018_148_MOESM1_ESM.docx]

**Additional file 1: Table S1.** Characteristics of participants involved in the six FGDs

| **FGD ID** | **Mother’s Age (years)** | **Child’s Age (months)** | **Education**  **Level** | **Mother’s Occupation** | **Recruitment Setting** | **FGD & No.** | **Mother’s Age (years)** | **Child’s Age (months)** | **Education**  **Level** | | **Mother’s Occupation** | **Recruitment Setting** |
| --- | --- | --- | --- | --- | --- | --- | --- | --- | --- | --- | --- | --- |
| **Focus group 1 - 10 August 2015** | | | | | | 3F | 27 | 6 | | High School | Worker | Khonkaen Hospital |
| 1A | 30 | 6 | High School | Factory worker | Khonkaen Hospital | **Focus group 4 - 19 August 2015** | | | | | | |
| 1B | 25 | 6 | Bachelor  degree | Secretary | Khonkaen Hospital | 4A | 33 | 4 | High School | | Seller | Khonkaen Hospital |
| 1C | 37 | 6 | High  school | Labourer | Khonkaen Hospital | 4B | 20 | 4 | Bachelor  degree | | Housewife | Khonkaen Hospital |
| 1D | 33 | 6 | Bachelor  degree | Seller | Khonkaen Hospital | 4C | 34 | 6 | Bachelor  degree | | Officer | Khonkaen Hospital |
| **Focus group 2 - 14 August 2015** | | | | | | 4D | 27 | 6 | | Bachelor  degree | Own Business | Private Hospital |
| 2A | 20 | 4 | Bachelor  degree | Housewife | Numphong Hospital | 4E | 20 | 6 | High School | | Labor | Khonkaen Hospital |
| 2B | 20 | 4 | High School | Housewife | Numphong Hospital | 4F | 33 | 6 | Bachelor  degree | | Own Business | Khonkaen Hospital |
| 2C | 35 | 4 | Bachelor  degree | Seller | Numphong Hospital | **Focus group 5 - 20 August 2015** | | | | | | |
| 2D | 23 | 6 | Bachelor  degree | Housewife | Numphong Hospital | 5A | 34 | 6 | Master  degree | | Doctor | Private Hospital |
| 2E | 36 | 4 | High School | Housewife | Numphong Hospital | 5B | 37 | 4 | Bachelor  degree | | Nurse | Private Hospital |
| 2F | 24 | 4 | High School | Factory worker | Numphong Hospital | 5C | 30 | 6 | Bachelor  degree | | Analyst | Private Hospital |
| **Focus group 3 - 17 August 2015** | | | | | | 5D | 37 | 4 | | Master  degree | Lecturer | Private Hospital |
| 3A | 35 | 6 | Bachelor  degree | Seller | Khonkaen Hospital | 5E | 34 | 4 | Bachelor  degree | | Housewife | Private Hospital |
| 3B | 34 | 4 | Bachelor  degree | Librarian | Khonkaen Hospital | **Focus group 6 - 22 August 2015** | | | | | | |
| 3C | 32 | 6 | Bachelor  degree | Pharmacist | Private Hospital | 6A | 28 | 4 | Bachelor  degree | | Nurse | Khonkaen Hospital |
| 3D | 34 | 4 | Bachelor  degree | IT staff | Private Hospital | 6B | 30 | 4 | Master  Degree | | Lecturer | Khonkaen Hospital |
| 3E | 30 | 6 | Bachelor  degree | Secretary | Khonkaen Hospital | 6C | 30 | 4 | Bachelor  degree | | Nurse | Khonkaen Hospital |
